# Keratosis lichenoides chronica: A case report and focused overview of the literature

**DOI:** 10.1111/ajd.13713

**Published:** 2021-09-13

**Authors:** Italo Francesco Aromolo, Serena Giacalone, Giovanni Genovese, Carlo Alberto Maronese, Angelo Valerio Marzano

**Affiliations:** ^1^ Dermatology Unit Fondazione IRCCS Ca’ Granda Ospedale Maggiore Policlinico Milan Italy; ^2^ Department of Pathophysiology and Transplantation Università degli Studi di Milano Milan Italy

Dear Editor,

Keratosis lichenoides chronica (KLC) is an inflammatory skin disorder, characterised by diffuse, lichenoid hyperkeratotic papules arranged in an either linear or reticular fashion, erythematosquamous plaques and seborrheic‐like dermatitis on the face.^1^ KLC runs a chronic, progressive course and demonstrates poor response to treatment.^1^, ^2^ Herein, we report the case of a 55‐year‐old woman referred to our Dermatology Unit for a 4‐year history of diffuse, persistent, slightly pruritic cutaneous lesions, predominantly affecting her face and limbs. Previously, she had been diagnosed with *pityriasis lichenoides chronica* and then with discoid lupus erythematosus. Topical corticosteroids, calcineurin inhibitors, phototherapy, hydroxychloroquine and methotrexate had been tried with little to no benefit. Physical examination revealed numerous, 2–4 mm diameter, keratotic, red‐purplish papules symmetrically distributed to the upper and lower limbs and arranged in a linear‐reticular pattern. On the face, there were well‐demarcated erythematous papules and plaques with yellowish scales, especially in seborrheic areas. Cutaneous lesions were reported to improve with sunlight exposure. Concomitant blepharitis with a painful erosion on the tarsal conjunctiva of the left eye was an additional finding. Finally, a background photodistributed erythema was documented (Fig. 1). No involvement of scalp or oral and genital mucosae was recorded. Laboratory examinations were within normal ranges. Histopathological examination revealed focal epidermal parakeratosis and hypergranulosis, band‐like lymphocytic infiltrates in the dermis and exocytosis of lymphocytes at the dermal–epidermal junction (Fig. 2). Direct immunofluorescence showed granular Immunoglobulin M (IgM) deposits at the dermal–epidermal junction and within dermal papillae. Based on clinical and histological features, a diagnosis of *keratosis lichenoides chronica* was made. The patient refused testing for nucleotide‐binding domain and leucine‐rich repeat containing proteins 1 (NLRP1) gene.

**Figure 1:**
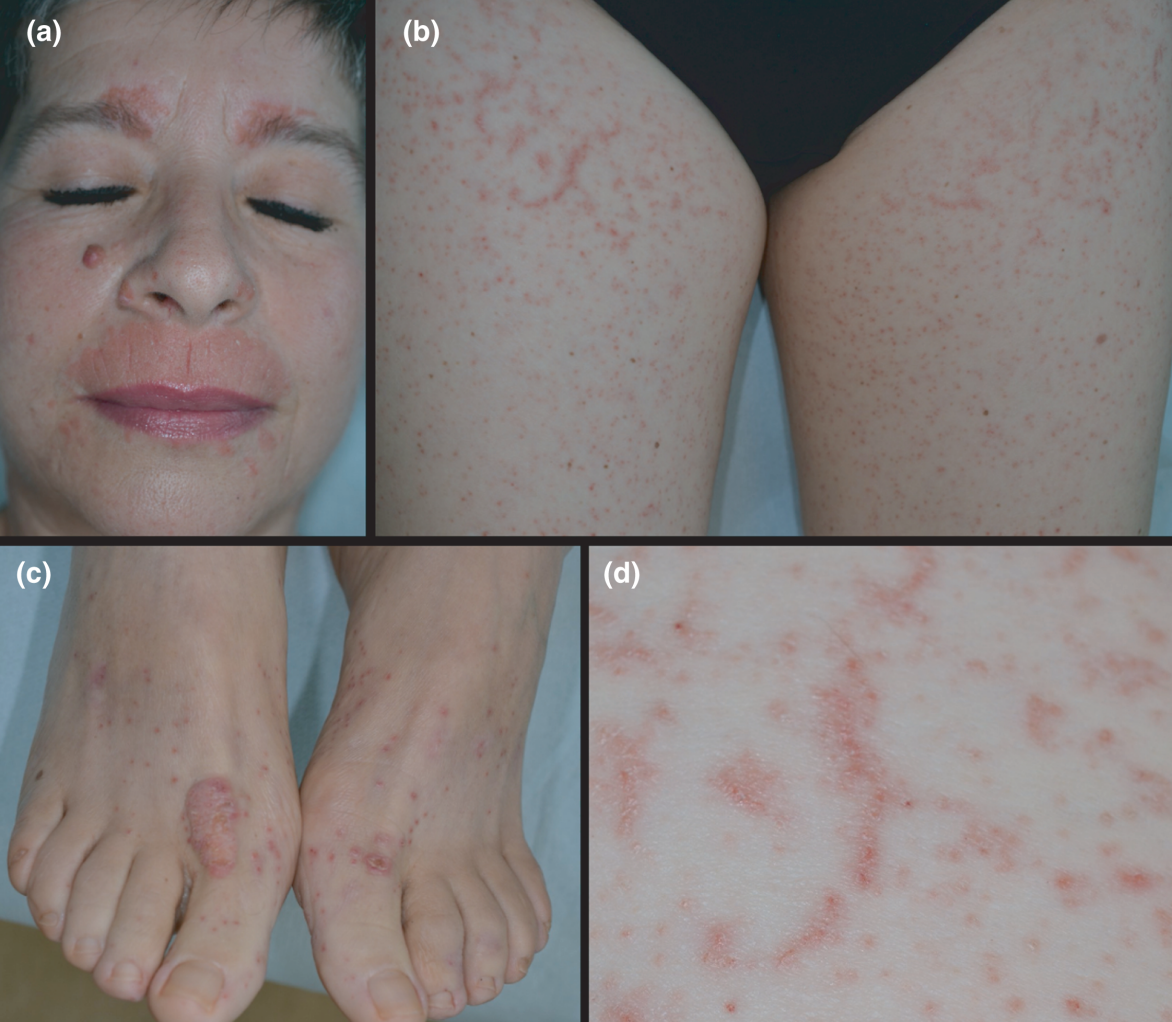
Typical KLC clinical features in a 55‐year‐old woman: (a) erythema and scaling in seborrheic areas; (b) red, keratotic papules arranged in a linear‐reticular pattern; (c) a verrucous plaque on dorsal feet surface; (d) close up view of lesions localised on the right thigh.

**Figure 2:**
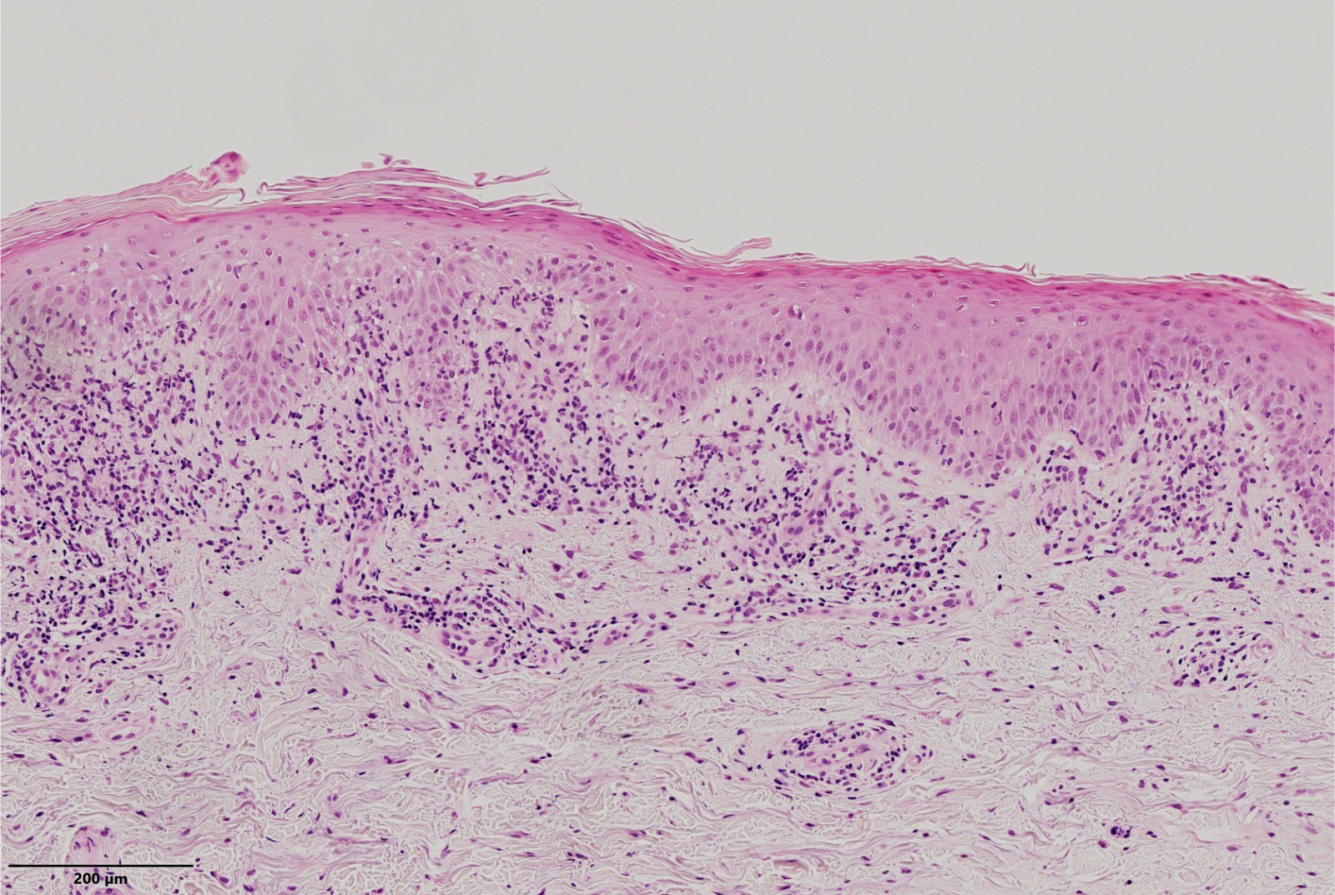
Histopathological examination revealed presence of epidermal focal parakeratosis, hypergranulosis and lymphocytic band‐like infiltrates in the dermis, with exocytosis of lymphocytes at the dermal–epidermal junction (Haematoxylin & Eosin, X20).

Also known as Nekam’s disease, KLC is an exceptionally rare dermatosis, with only 82 cases reported to date (Table S1). It shows a peak of incidence in the fourth decade, with a slight male predominance. About 25% of cases are paediatric. KLC is hallmarked by asymptomatic, violaceous, scaly papules arranged in a reticular pattern over the body, more commonly on the limbs, and showing either seborrheic dermatitis‐like or rosacea‐like features on the face.^1^ Palmoplantar keratoderma may also occur. Less frequently, oral and genital mucosae can be affected with ulcerative lesions, while ocular involvement usually manifests with blepharitis, conjunctivitis, uveitis or iridocyclitis. Cutaneous appendages can be involved as well, with onychodystrophy or alopecia.^2^ Histopathological findings typically include hyperkeratosis with focal parakeratosis, irregular acanthosis admixed with areas of atrophy, vacuolar degeneration of the basal layer and mixed inflammatory lichenoid infiltrates in the upper dermis (often around infundibula and acrosyringia).^3^ Further complicating its diagnosis, several clinicopathological variants of KLC have been described, including vascular, purpuric, lupus‐like, porokeratotic associated to amyloidosis and generalised forms (Table S2).

Existing therapeutic options include systemic retinoids (acitretin, isotretinoin and etretinate) and phototherapy (psoralen‐UVA, narrow band‐UVB), which can lead to complete remission in almost half of patients, alone or in combination. Systemic steroids, antimalarials and antibiotics showed minimal effectiveness.^2^

The pathogenesis of KLC has not yet been elucidated fully. Traditionally, KLC was identified as a variant of LRP, and was long presumed to have the same underlying immune‐mediated pathophysiology.^3^, ^4^ Beyond clinical and histopathological differences, reclassification of KLC as a separate entity was recently substantiated by the discovery of a gain‐of‐function mutation in nucleotide‐binding domain and leucine‐rich repeat containing proteins 1 (NLRP1) gene in a family with semi‐dominantly inherited KLC.^5^ NLRP1 is an inflammasome sensor protein highly expressed in keratinocytes and cutaneous fibroblasts. In response to a variety of stimuli, NLRP1 activates caspase‐1 leading to increased production of interleukin (IL)‐1 and IL‐18. The KLC‐causing mutation disrupts a leucin‐rich repeat (LRR) domain, resulting in constitutive NLRP1 self‐dimerisation and inflammasome activation.^5^ Such autoinflammatory setting determines reactive keratinocyte proliferation, which translates into parakeratotic hyperkeratosis on histology.^1^, ^5^ Over time, excessive keratinocyte turnover may favour the rise of neoplastic skin lesions, such as multiple keratoacanthomas.^5^, ^6^

Accordingly, familiar KLC was included within the spectrum of autoinflammatory keratinisation diseases, an umbrella term encompassing monogenic skin diseases with an autoinflammatory pathogenesis.^7^ Germinal NLRP1 gene mutations have also been described in patients with dyskeratotic skin manifestations (e.g. follicular hyperkeratotic papules), polyarthritis and recurrent fever: suitably, this newly discovered disorder was named NLRP1‐associated autoinflammation with arthritis and dyskeratosis (NAIAD).^8^

Such novel insights into familiar KLC may pave the way for anti‐IL‐1 therapeutic trials also in sporadic cases. Indeed, there are no reports on IL‐1 receptor antagonist (i.e. anakinra) treatment in familiar KLC, although good response to anakinra was recorded in a case of NAIAD.^8^

Further research will be needed to better understand the pathophysiology of both familiar and sporadic KLC cases.

## Details of contribution of individual authors

Italo Francesco Aromolo, Serena Giacalone, Giovanni Genovese and Carlo Alberto Maronese equally participated in data acquisition, analysis, interpretation and drafting of the manuscript. Angelo Valerio Marzano participated in study concept and design and supervised the study. All authors critically revised the manuscript for important intellectual content and approved the final manuscript.

## Patient consent statement

Written informed consent was obtained from the patient included in the study, regarding also the publication of the photos.

## Ethics approval

The study was conducted in accordance with the ethical standards of the responsible committee on human experimentation (institutional and national), with the Helsinki Declaration of 1975, as revised in 2000, and with the Taipei Declaration.

## Supporting information

Table S1. Demographics and clinical features of the 82 published cases of KLC^1‐10^.

Table S2. Clinical and histopathological features of reported KLC variants.

## Data Availability

Anonymised data will be shared upon reasonable request from any qualified investigator for purposes of replicating procedures and results.
