# Supplementary material for: Keratosis lichenoides chronica: A case report and focused overview of the literature
Source: Australas J Dermatol. 2021 Sep 13;63(1):e99–e102. doi: 10.1111/ajd.13713 (PMC9291015; doi:10.1111/ajd.13713)
Supplement: Supplementary file 1 — Table S1. Demographics and clinical features of the 82 published cases of KLC1‐10. [file AJD-63-e99-s001.docx]

**Supplementary Table 1**

|  |  |
| --- | --- |
| **Epidemiology** |  |
| Mean age at diagnosis, years (range) | 36.5 (0.5-78) |
| Pediatric cases, n (%) | 20 (24.4) |
| Male to female ratio, n (%) | 51 (1.64) |
| **Localization of cutaneous lesions,** n (%) |  |
| Trunk | 48 (58.5) |
| Upper limbs | 74 (90.2) |
| Lower limbs | 70 (85.3) |
| Face | 67 (81.7) |
| Palmoplantar areas | 21 (25.6) |
| Buttocks | 21 (25.6) |
| Neck | 6 (7.3) |
| **Other clinical findings**, n (%) |  |
| Alopecia | 7 (8.5) |
| Onychodystrophy | 17/71* (24) |
| Oral lesions | 19/72* (26.4) |
| Ocular lesions | 13/68* (19.2) |
| Genital lesions | 1. (22) |

*not all data were always available for each reported case.

**Supplementary Table 1**. Demographics and clinical features of the 82 published cases of KLC^1-10^.

**References**

1. Pistoni F, Peroni A, Colato C *et al.* Keratosis lichenoides chronica: Case-based review of treatment options. *J Dermatolog Treat.* 2016 Aug;27(4):383-8.
2. Aruna C, Ramamurthy DV, Neelima T *et al.* Nekam's disease. *Indian Dermatol Online J.* 2016;7(6):520-522.
3. Julien B, Pierre K, El-Haber C *et al.* Two Cases of Pediatric Keratosis Lichenoides Chronica with Review of the Clinical and Hitopathological Features of Pediatric versus Adult Presentation and Treatment with Acitretin. *J Clin Exp Dermatol.* 2019 Res 10:476.
4. Li AW, Damsky W, King BA. Keratosis lichenoides chronica successfully treated with isotretinoin and methotrexate. *JAAD* Case Rep. 2017 14;3(3):205-207.
5. Escanilla C, Truffello D, Cevallos C *et al.* Keratosis lichenoides chronica: First case reported in Chile. *Dermatol Online J.* 2019 15;25(5):13030/qt6jc473s4.
6. Barisani A, Savoia F, Sgubbi P *et al.* Keratosis lichenoides chronica with an atypical clinical presentation and variable histopathological features. *J Dtsch Dermatol Ges.* 2016;14(11):1136-1139.
7. Nasimi M, Azizpour A, Lajevardi V *et al.* A case of pediatric keratosis lichenoides chronica with unusual presentation of severe oral erosions. *JAAD* Case Rep. 2018 6;4(3):267-269.
8. Geng S, Liu Y, Wang H et al. Hypertrophic Lichenoid Eruption in a Child Successfully Treated Using Acitretin and Surgery: A Case Report and Literature Review. *Pediatr Dermatol*. 2015;32(6):e238-41.
9. Santesteban-Muruzábal R, Larrea-García M, Yanguas-Bayona I. Generalized Keratosis Lichenoides Chronica. *Actas Dermosifiliogr.* 2016;107(3):249. English, Spanish.
10. Dogan S, Kılınç E, Özaygen GE *et al.* Childhood-Onset Keratosis Lichenoides Chronica Accompanied by Severe Hair Loss. *Skinmed.* 2017 1;15(3):211-213.
