# Supplementary material for: Keratosis lichenoides chronica: A case report and focused overview of the literature
Source: Australas J Dermatol. 2021 Sep 13;63(1):e99–e102. doi: 10.1111/ajd.13713 (PMC9291015; doi:10.1111/ajd.13713)
Supplement: Supplementary file 2 — Table S2. Clinical and histopathological features of reported KLC variants. [file AJD-63-e99-s002.docx]

**Supplementary Table 2**

| **KLC Variants** | **Clinical specificities** | **Histological specificities** | **Additional findings** |
| --- | --- | --- | --- |
| Vascular ^1,2^ | Papules on telangiectatic background, with moderate to severe itch | Dilated dermal blood vessels | No response to systemic retinoids of vascular component; very prolonged diagnostic delay (>20 years) |
| Purpuric ^3,4^ | Marked purpuric papules, preferentially at face | Erythrocyte extravasation | More common in children |
| Lupus-like ^5,6,7^ | Facial erythemato-keratotic plaques | Numerous necrotic keratinocytes, mucin deposits | Less responsive to systemic retinoids |
| Porokeratotic associated to amyloidosis ^8^ | Classic presentation, but more severe | Focal area of porokeratosis and focal, congo red positive, eosinophilic material |  |
| Generalized ^9^ | Lesions involving almost the entire body surface | None |  |

**Supplementary Table 2**. Clinical and histopathological features of reported KLC variants.

**References**

1. David M, Filhaber A, Rotem A *et al.* Keratosis lichenoides chronica with prominent telangiectasia: response to etretinate. *J Am Acad Dermatol.* 1989;21(5 Pt 2):1112-4.
2. Nijsten T, Mentens G, Lambert J. Vascular variant of keratosis lichenoides chronica associated with hypothyroidism and response to tacalcitol and acitretin. *Acta Derm Venereol.* 2002;82(2):128-30.
3. Ruiz-Maldonado R, Duran-McKinster C, Orozco-Covarrubias L *et al*. Keratosis lichenoides chronica in pediatric patients: a different disease? *J Am Acad Dermatol.* 2007;56(2 Suppl):S1-5.
4. García-Salces I, Güezmes A, Moro F. Variante purpúrica de queratosis liquenoide crónica [Purpuric variant of keratosis lichenoides chronica]. *Actas Dermosifiliogr.* 2010;101(3):272-3.
5. Arata J, Seno A, Tada J *et al.* Peculiar facial erythematosquamous lesions in two siblings with cyclical summer improvement and winter relapse: a variant of keratosis lichenoides chronica? *J Am Acad Dermatol.* 1993;28(5 Pt 2):870-3.
6. Wee JS, Viros A, Ffolkes L *et al.* Keratosis lichenoides chronica masquerading as discoid lupus erythematosus. *Clin Exp Dermatol.* 2013;38(3):327-9.
7. Barisani A, Savoia F, Sgubbi P *et al.* Keratosis lichenoides chronica with an atypical clinical presentation and variable histopathological features. *J Dtsch Dermatol Ges.* 2016;14(11):1136-1139.
8. Stefanato CM, Youssef EA, Cerio R *et al.* Atypical Nekam's disease--keratosis lichenoides chronica associated with porokeratotic histology and amyloidosis. *Clin Exp Dermatol.* 1993;18(3):274-6.
9. Santesteban-Muruzábal R, Larrea-García M, Yanguas-Bayona I. Generalized Keratosis Lichenoides Chronica. *Actas Dermosifiliogr.* 2016;107(3):249. English, Spanish.
